# Alcohol use and extramarital sex among men in Cameroon

**DOI:** 10.1186/1472-698X-7-6

**Published:** 2007-08-03

**Authors:** Eugene J Kongnyuy, Charles Shey Wiysonge

**Affiliations:** 1 Child and Reproductive Health Group, Liverpool School of Tropical Medicine, Liverpool, UK; 2 South African Cochrane Centre, Medical Research Council, Cape Town, South Africa

## Abstract

**Background:**

The spread of HIV in sub-Saharan Africa is believed to be driven by unsafe sex, and identification of modifiable risk factors of the latter is needed for comprehensive HIV prevention programming in the region. Some previous studies suggest an association between alcohol abuse and unsafe sexual behaviour, such as multiple concurrent sexual partnerships and inconsistent condom use in sex with non-spousal non-cohabiting partners. However, most of these studies were conducted in developed countries and the few studies in Africa were conducted among well-defined social groups such as men attending beer halls or sexually transmitted infection clinics. We therefore examined the association between alcohol and extramarital sex (a sign of multiple concurrent sexual partnerships) among men in a population-based survey in Cameroon; a low-income country in sub-Saharan Africa with a high rate of alcohol abuse and a generalised HIV epidemic.

**Methods:**

We analyzed data from 2678 formally married or cohabiting men aged 15 to 59 years, who participated in the 2004 Cameroon Demographic and Health Survey, using a multivariate regression model.

**Results:**

A quarter of the men (25.8%) declared having taken alcohol before their last sexual intercourse and 21% indicated that the last sex was with a woman other than their wife or cohabiting partner. After controlling for possible confounding by other socio-demographic characteristics, alcohol use was significantly associated with having extramarital sex: adjusted odds ratio (OR) 1.70, 95% confidence intervals (CI) 1.40 to 2.05. Older age (30–44 years: OR 3.06, 95%CI 2.16–4.27 and 45–59 years: OR 4.10, 95%CI 2.16–4.27), higher education (OR 1.25, 95%CI 1.10–1.45), and wealth (OR 1.71, 95%CI 1.50–1.98) were also significantly associated with higher odds of having extramarital sex. The men were more likely to have used a condom in their last sex if it was extramarital (OR 10.50, 95%CI 8.10–13.66). Older age at first sex (16–19 years: OR 0.81, 95%CI 0.72–0.90 and > 19 years: OR 0.74, 95% CI 0.65–0.87) and being the head of a household (OR 0.17, 95%CI 0.14–0.22) significantly decreased the odds of having sex outside of marriage. Religion and place of residence (whether urban or rural) were not significantly associated with extramarital sex.

**Conclusion:**

Alcohol use is associated with having multiple concurrent non-spousal sexual partnerships among married men in Cameroon. We cannot infer a causal relationship between alcohol abuse and unsafe sex from this cross-sectional study, as both alcohol use and unsafe sexual behaviour may have a common set of causal personal and social factors. However, given the consistency with results of studies in other settings and the biologic plausibility of the link between alcohol intake and unsafe sex, our findings underscore the need for integrating alcohol abuse and HIV prevention efforts in Cameroon and other African countries with similar social profiles.

## Background

Previous studies suggest an association between alcohol intake and unsafe sexual behaviour [1-4]. Alcohol lowers the cognitive reserve such that people who abuse alcohol are more likely to engage in unprotected sex even with a high risk partner [5]. Corte and Sommers confirmed this association in a recent review, and postulate that alcohol leads to unsafe sex only among persons who have sexual expectancies about the effects of alcohol prior to drinking [6]. This would imply that alcohol leads to unsafe sex for people who have the perception that alcohol will enhance sex or give them courage to approach their sex partners; but have no effect in people who do not have such preconceived ideas. However, it is uncertain whether this hypothesis explains why some studies fail to find an association between alcohol use and unsafe sexual behaviours [7,8].

Most research on the relationship between alcohol use and sexual behaviour has been largely limited to developed countries [9-11], with few studies conducted in sub-Saharan Africa [12-14]. The few studies conducted in sub-Saharan Africa (mostly, in well-defined social settings) have found significant associations between alcohol use and multiple concurrent sexual partnerships and inconsistent condom use with casual partners and sex workers [12-14]. Elsewhere, a study of Vietnamese adolescents found that alcohol use was significantly associated with both engagement in sexual activity and the intention to engage in sexual activity [15].

Research has focused mainly on adolescents, minority groups, migrants, youths, secondary schools and university students [16-20]. Like many other areas of research, men have been largely ignored. Few studies conducted on men have focused on specific groups such as men who have sex with men, men attending beer halls, and truck drivers [21,22], and how alcohol can affect marital life [23]. In a prospective cohort of 14,127 participants in the United States, drinking was found to decrease with age and married men were less likely to drink than unmarried men [24].

Cameroon is a country characterized by both high alcohol intake and high prevalence of HIV [25]. Alcohol consumption in Cameroon is estimated to be 2.6 litres of pure alcohol per capita for men and women older than 15 years [26]. About 41.4% of men and 25.8% of women consume alcohol regularly in Cameroon [26]. The usual diet in Cameroon is rich in alcohol content and excessive alcohol intake is a common finding [27]. Alcohol is consumed for varied reasons, including social, religious, and even medicinal. The trend of drinking patterns is not documented, but applying evidence from elsewhere we expect new drinking patterns to build on old traditional drinking behaviours along with other social changes [28]. The overall HIV prevalence among resident adult Cameroonians aged 15–49 years is about 5.5 %. The prevalence is higher in women (6.8%) than in men (4.1%) [25]. The HIV prevalence varies by marital status: 3.5% among unmarried, 6.2% among married and 18.5% among formerly married (widowed or divorced) persons. The prevalence in monogamous and polygamous families is 6.6% and 5.5% respectively. To sustain a heterosexual HIV epidemic, an (infected) person must have unprotected sex with more than one partner. The only way this can happen in marriage is through extramarital sexual relationships, given that polygamy is not a significant HIV risk factor in our setting. Since alcohol is the most common form of substance abuse in Cameroon [5,26] and is associated with unsafe sexual behaviours in other societies as indicated above, it may be a common and potentially modifiable risk factor for HIV infection in the country. We therefore examined the association between alcohol use and extramarital sex, because men who have such unsafe sexual behaviours are (potential) key agents for the transmission of HIV and other sexually transmitted infections. Although this study focuses on married men, single men who have multiple concurrent sex partners are equally at risk of acquiring HIV infection and (if already infected) of transmitting same to their sex partners.

## Methods

### Design

This cross-sectional and population-based study uses data from the 2004 Cameroon Demographic and Health Survey (DHS). Since the details of the survey methods are published else where [25,29], we provide only a brief description here. Following informed consent, structured interviews were used to obtain information from men aged 15 to 49 years about various socio-demographic characteristics including current sexual activity and alcohol use. The survey was approved by the Ethics Committee of the ORC Macro at Calverton in the USA and by the National Ethics Committee in the Ministry of Health in Cameroon. All information was collected confidentially. We obtained the data from ORC Macro, and have included only married and cohabiting men in our analysis.

### Population and sampling

The survey used a two-stage cluster sampling technique. The country was stratified into 12 domains (i.e. the 10 provinces and the two major cities of Yaoundé and Douala). The provinces were further stratified into urban and rural areas. Each domain was composed of clusters called "enumeration areas", which were established in the 2003 General Population and Housing Census. Within each domain, a two-stage sample was selected. The first stage consisted of selecting 466 clusters (primary sampling units) with the probability of selection proportional to the size; the size being the number of households in the cluster. The second stage involved the systematic sampling of households from the selected clusters. All men aged 15 to 59 years in the selected households were interviewed.

### Variables

We selected extramarital sex as the dependent variable and alcohol use as the main independent variable, with potential confounding variables being age of the man, whether the man was the head of the household (or not), level of education, place of residence, age at first sex, socio-economic status, and religion. We defined the variables as follows:

#### Married men

Married men as used in this study include all married and cohabiting men.

#### Extramarital sex

We defined extramarital sex as the act of having sexual intercourse with another woman other than one's spouse or cohabitating sex partner. Co-habitation or free union is common in Cameroon and some couples have long lasting stable unions without formal marriage (legal or traditional). We excluded all men who were not married or cohabiting from our analysis.

#### Alcohol use

Alcohol use was defined as the act of drinking alcohol before the last sexual intercourse. The amount of alcohol taken by the participant was not quantified. But rather, four categories were identified, namely (a) neither the respondent nor his partner took alcohol before the last sex, (b) the respondent took alcohol but the sex partner did not, (c) the sex partner took alcohol but the respondent did not, and (d) both the respondent and his sex partner took alcohol.

#### Wealth index

We used the wealth index as a measure of socio-economic status. A score was given to each amenity according to the Health, Nutrition and Population/Poverty Thematic Group of the World Bank [30]. The total score for each household constituted the wealth index score for that household. Each man was assigned the wealth index score of his household [29]. The total score was converted to an ordinal scale with three equal-sized categories based on percentiles of wealth score: < 33.33^th ^percentile (poor), 33.33th to 66.66^th ^percentile (middle), and > 66.66^th ^percentile (rich).

#### Other variables

Educational attainment was defined as never been to school, primary, and secondary or higher education; age was stratified into three 15-year age bands (15–29 years, 30–44 years, and 45–59 years); place of residence was defined as rural or urban; and religion as Christians, Muslims, and others.

### Statistical analyses

All cases in the DHS data are given weights to adjust for differences in probability of selection and to adjust for non-response in order toproduce the proper representation [25]. We used individual weights for statistical analyses in this study, using SPSS version 13.0 for Windows. In the first step of the analyses we described the general characteristics of the study population. We have presented the results as percentages for categorical variables and means (standard deviations) for continuous variables. Secondly, we conducted univariate analyses to examine bivariate associations between each independent variable and extramarital sex using the Chi-square test. Lastly, we constructed a multiple regression model to estimate the relationship between alcohol use and extramarital sex, controlling for potential confounders. The variables entered into the multiple regression model were categorized as follows: age (15–29 years, 30–44 years and 45–59 years), education (primary or less versus secondary or higher), wealth index (low versus high), residence (rural versus urban), religion (Christians versus others), age at sexual debut (< 16 yrs, 16–19 years, and > 19 yrs), head of household (yes versus no), drank alcohol before last sexual intercourse (yes versus no), and condom use during the last sexual intercourse (yes versus no). The cut-off point for wealth index was the median for wealth index variable. All significance tests were two-tailed and statistical significance was defined at the 5% alpha level.

## Results

### Socio-demographic characteristics of the study population

A total of 2678 married men aged 15 to 59 years were included in the analysis. The mean age was 37.0 years (standard deviation [SD] 10.5) and the mean age at first intercourse was 18.6 years (SD 4.0). Table 1 shows the socio-demographic characteristics of the study participants. Most participants (45.2%) were aged 30 to 44 years. About 11.5% of the participants had never been to school while 45.5% had at least secondary education. They were evenly distributed between the rural and urban areas: 47.6% and 52.4%, respectively. About two-thirds (67.2%) of the participants were Christians while Muslims constituted almost one-fifth (19.5%). Most of the men were family heads (83.2%), while the rest had different relationships to the family head such as son, son-in-law, grand son, brother, and adopted child. About one-quarter (25.8%) of the men declared that they took alcohol before their last sexual intercourse and one out of five (21%) indicated that his last sex was with a woman other than his wife or cohabiting partner.

**Table 1:** Percentage distribution of married Cameroonian men by selected characteristics, Cameroon Demographic and Health Survey 2004

Characteristic	Percentage
**Age (years)**	
15–29	28.2
30–44	45.2
45–59	26.6
**Education**	
No school	16.9
Primary	37.6
Secondary or higher	45.5
**Residence**	
Rural	47.6
Urban	52.4
**Religion**	
Christians	67.2
Muslims	19.5
Others	13.3
**Age at first intercourse (years)**	
< 16	18.7
16–19	46.5
> 19	34.4
**Head of household**	
Yes	83.2
No	16.8
**Drank alcohol before last intercourse**	
No	73.3
Yes	26.7

### Univariate analyses

We present the unadjusted odds ratios (OR) and their 95% confidence intervals (CI) for the bivariate association between extramarital sex and each independent variable in Table 2. The first category of each independent variable is taken as the reference category (and given an OR of 1.00) and the effect of the other categories on extramarital sex is compared with this reference category.

**Table 2:** Bivariate association between extramarital sex and selected characteristics of married Cameroonian men, 2004

Characteristic	Extramarital sex		OR (95%CI)	p-value
	**No**	**Yes**		
**Age (years)**				
15–29	490	266	1	-
30–44	1001	206	8.95 (7.24–11.06)	< 0.001
45–59	626	87	13.25 (10.13–17.38)	< 0.001
**Education**				
No school	418	35	1	-
Primary	802	204	3.04 (2.10–4.48)	< 0.001
Secondary or higher	897	323	4.30 (3.00–6.29)	< 0.001
**Wealth index**				
Poor	794	98	1	-
Middle	674	220	2.64 (2.03–3.46)	< 0.001
Rich	649	244	3.34 (2.34–3.98)	< 0.001
**Residence**				
Rural	1087	189	1	-
Urban	1029	373	2.08 (1.72–2.54)	< 0.001
**Religion**				
Christians	1379	420	1	-
Muslims	443	80	0.59 (0.45–0.77)	< 0.001
Others	294	62	0.69 (0.51–0.93)	0.014
**Age at first intercourse (years)**				
< 16	346	155	1	-
16–19	956	289	0.67 (0.54–0.85)	0.001
> 19	809	114	0.31 (0.24–0.41)	< 0.001
**Head of the household**				
No	229	221	1	-
Yes	1888	340	0.19 (0.15–0.23)	< 0.001
**Drank alcohol before the last intercourse**				
None	1540	323	1	-
Respondent only	373	88	1.12 (0.86–1.47)	0.378
Partner only	31	8	1.23 (0.48–2.27)	0.605
Both respondent and partner	169	62	1.75 (1.25–2.42)	< 0.001
**Condom use during the last intercourse**				
No	2025	225	1	
Yes	158	270	14.28 (12.50–20.00)	< 0.001

OR, odds ratio; 95%CI, 95% confidence intervals

The odds of having extramarital sex increased with age, educational attainment, and wealth index (all p < 0.001). In addition, men resident in urban areas were twice as likely as rural men to have extramarital sex (OR 2.08, 95%CI 1.72 to 2.54). Similarly, condoms were more likely to be used in extramarital sex than in sex within marriage (OR 14.28, 95%CI 12.50 to 20.00). Compared to Christians, Muslims were less likely to engage in extramarital sex (OR 0.59, 95%CI 0.45 to 0.77). Family heads were equally less likely to engage in extramarital sex (OR 0.19, 95%CI 0.15 to 0.23). Older age at sexual debut also decreased the odds of having extramarital sex: OR 0.67 (95% CI 0.54 to 0.85) for age 16–19 years and OR 0.31 (95% CI 0.24 to 0.41) for age 19 years or older, compared to less than 16 years.

On examining the effect of alcohol use before the last sexual intercourse, we found that if only the respondent or his partner took alcohol there was no significant association with having extramarital sex. However, when both partners took alcohol the odds of having extramarital sex increased almost two-fold (p < 0.001).

### Multivariate analyses

We constructed a multiple regression model based on the factors identified in univariate analyses as having significant associations with extramarital sex. The independent variables entered into the model were age, education, wealth, residence, religion, age at first sex, condom use during last sex, and alcohol intake before last sex. Alcohol use was reduced to two categories according to whether or not the respondent had taken alcohol himself before the last sexual intercourse, irrespective of whether his partner also took alcohol.

Table 3 shows the results of these multiple regression analyses. Alcohol use was significantly associated with increased odds of having extramarital sex, after controlling for potential confounding by age, head of household status, education, religion, age of sexual debut, wealth, and condom use (OR 1.70, 95%CI 1.40 to 2.05).

**Table 3:** Factors associated with extramarital sex in Cameroonian men identified by multiple regression analysis

Characteristic	Odds ratio	95% CI	p-value
**Drank alcohol before last intercourse**			
*no*	1	-	-
*yes*	1.70	1.40–2.05	< 0.001
**Head of household**			
*no*	1	-	-
*yes*	0.17	0.14–0.22	< 0.001
**Man's age**			
*15–29 yrs*	1	-	-
*30–44 years*	3.06	2.16–4.27	< 0.001
*45–59 years*	4.10	3.50–5.71	< 0.001
**Education**			
*primary or less*	1	-	-
*secondary or higher*	1.25	1.10–1.45	< 0.001
**Religion**			
*Christians*	1	-	-
*Others*	0.90	0.81–1.04	0.086
**Age of first intercourse**			
*< 16 yrs*	1	-	-
*16–19 years*	0.81	0.72–0.90	< 0.001
*> 19 years*	0.74	0.65–0.87	< 0.001
**Wealth index**			
*low*	1	-	-
*high*	1.71	1.50–1.98	< 0.001
**Residence**			
*rural*	1	-	-
*urban*	1.13	0.90–1.42	0.245
**Condom use during last intercourse**			
*no*	1	-	-
*yes*	10.50	8.10–13.66	< 0.001

CI, confidence intervals

Other characteristics which were independently associated with increased odds of having extramarital sex included age (30–44 years: OR 3.06, 95%CI 2.16 to 4.27 and 45–59 years: OR 4.10, 95%CI 2.16 to 4.27), higher education (OR 1.25, 95%CI 1.10 to 1.45), and wealth (OR 1.71, 95%CI 1.50 to 1.98). In addition, the odds of using a condom increased during extramarital sex (OR 10.50, 95% CI 8.10 to 13.66). Men who were heads of household were less likely to engage in extramarital sex (OR 0.17, 95%CI 0.14 to 0.22), just like men who first sex when they were older than 16 years (16–19 years: OR 0.81, 95%CI 0.72 to 0.90 and > 19 years: OR 0.74, 95% CI 0.65–0.87). However, after controlling for confounding by other factors, religion (p = 0.086) and place of residence (p = 0.245) were no longer significantly associated with having extramarital sex.

Many predictor variables were significantly correlated with each other. Man's age was negatively correlated to age at sexual debut (p < 0.001). Religion was positively correlated to age at sexual debut (p < 0.001). Education was positively correlated to religion (p < 0.001), age at sexual debut (p < 0.001), and wealth index (p < 0.001). However, there was no significant correlation between condom use and drinking alcohol before sex (p = 0.40), despite the fact that both factors were significantly associated with extramarital sex.

## Discussion

We observed that drinking alcohol significantly increased the odds of extramarital sex in Cameroon. Overall, 21% of our participants reported that their last sexual intercourse was outside of marriage. This is higher than what has been reported in other African countries, for example, Mitsunaga and colleagues reported in 2003 that 11% of men have extramarital sex within a year in Nigeria [31]. The differences in the prevalence of extramarital sex may either reflect differences in sexual behaviours in different populations [32,33] or be due to the fact that we included cohabiting relationships in our study. Cohabitation is a common feature in Cameroon and sometimes cohabiting couples never get formally married throughout their lives. All the studies, however, prove that extramarital sex remains common despite being an unacceptable practice in almost all societies. In a survey in Australia, most people agreed that premarital sex was acceptable, that oral sex was considered normal sex, that sex was important for a sense of well-being, and that extramarital sex was unacceptable [34].

The association between alcohol intake and unsafe sex has been reported elsewhere. Gibney and colleagues reported an association between alcohol use and having sex with commercial sex workers among truck drivers in Bangladesh in 2003 [22] and Tveit and collaborators reported less frequent use of condom among Norwegian men who combined alcohol intake and casual sex in 1996 [34]. More recently, Weiser and collaborators found in a population-based survey in Botswana that men who abuse alcohol were three to four times more likely to have multiple (concurrent) sex partners and unprotected sex and to engage transactional sex than non-drinkers [13].

Consistent with the findings of other studies [36-39], we found associations between several other factors and extramarital sex. Having first sexual intercourse before 16 years of age was a predictor of extramarital sex compared to having the first sexual intercourse after 16. In addition, older men were more likely to have extramarital sex than their younger counterparts. Being the head of a household also significantly decreased the odds of having extramarital sex. Four-fifths of our study population were heads of their households. In most African societies, men are the sole bread winners and the heads of their households. Men who were not heads of households were either related to the household head as a son, brother, son- in-law or other relatives. As the head of the household a man is probably less likely to engage in extra-spousal sex because he is more responsible and attached to his spouse(s) than men who are no heads of households.

We did not examine the effect of occupation on extramarital sex, although it has been reported in the literature [40]. However, we used other proxies of socio-economic status such as wealth index and level of education. Education and wealth index were both found to be significantly associated with having extramarital sexual relationships. This finding has not been consistent across the board [36,41]. While no association was found between socio-economic status and extramarital sexual relationships in Zambia [36] this was found to be the case in the United States of America [41]. The association between economic well being and multiple concurrent sexual relationships is to be expected because men with more resources have greater access to women than poorer men [42].

The present study has several limitations. Secondary analysis of data collected for a different purpose is limited by the data available, and information on some useful factors which could confound the relationship between alcohol use and extramarital sex may be lacking. In addition, men who have extramarital sex when drinking may also have lots of extramarital sex when not drinking. Furthermore, due to the cross-sectional nature of this study, the findings can only indicate associations and not causality [43] because unsafe sexual behaviour and alcohol use may have a common set of causal factors which are unknown or difficult to measure [44]. However, the consistency of study results across different settings [9-15,22,34], biologic plausibility [5,45], dose-response relationship [13], and strength of the association suggest that alcohol use is a cause rather than a consequence of unsafe sexual behaviour.

## Conclusion

Alcohol use is associated with having extramarital sex, a sign of multiple concurrent sexual partnerships, among married men in Cameroon. We cannot infer a causal relationship between alcohol abuse and unsafe sex from this cross-sectional study as both alcohol use and unsafe sexual behaviour may have a common set of causal personal and social factors. However, viewed in the context of the results of studies in other countries, the biologic plausibility and dose-response relationship between alcohol intake and unsafe sex, our findings underscore the need for integrating alcohol abuse and HIV prevention efforts in Cameroon and other countries in sub-Saharan Africa with similar social profiles.

## Competing interests

The author(s) declare that they have no competing interests.

## Authors' contributions

EJK conceived the study, extracted the data, did the analyses and interpretation, and wrote the first draft of the manuscript. CSW participated in the interpretation and critically revised the manuscript for important intellectual content. Both authors read and approved the final manuscript.

## Pre-publication history

The pre-publication history for this paper can be accessed here:



## References

[B1] Staton M, Leukefeld C, Logan T, Zimmerman R, Lynam D, Milich R, Martin C (1999). Risky sexual behavior and substance use among youth adults. Health Soc Work.

[B2] O'Hare T (2005). Risky sex and drinking contexts in freshmen first offenders. Addictive Behaviors.

[B3] Thompson JC, Kao T, Thomas RT (2005). The relationship between alcohol use and risk taking behavior in a large behavioral study. Preventive Medicine.

[B4] Abbey A, Saenz C, Buck PO, Parkhill MR, Hayman LW (2006). The effects of acute alcohol consumption, cognitive reserve, partner risk, and gender on sexual decision making. J Stud Alcohol.

[B5] Davies SJ, Pandit SA, Feeney A, Stevenson BJ, Kerwin RW, Nutt DJ (2005). Is there cognitive impairment in clinically 'healthy' abstinent alcohol dependence?. Alcohol Alcohol.

[B6] Corte CM, Sommers MS (2005). Alcohol and risky behaviors. Annu Rev Nurs Res.

[B7] Guo J, Chung I, Hill K, Hawkins J, Catalano R, Abbott R (2002). Developmental relationship between adolescent substance use and risky sexual behavior in youth adulthood. J Adolesc Health.

[B8] Kingree J, Betz H (2003). Risky sexual behavior in relation to marijuana and alcohol use among African-American, male adolescent detainees and their females. Drug Alcohol Depend.

[B9] Halpern-Felsher BL, Millstein SG, Ellen JM (1996). Relationship of alcohol use and risky sexual behavior: a review and analysis of findings. J Adolesc Health.

[B10] Hingson R, Heeren T, Winter MR, Wechler H (2003). Early age at first drunkenness as a factor in college students' unplanned and unprotected sex attributable to drinking. Pediatrics.

[B11] Stueve A, O'Donnell L, Duran R, San Doval A, Geier J (2002). Community Intervention Trial for Youth Study Team Being high and taking sexual risks: findings from a multisite survey of urban young men who have sex with men. AIDS Educ Prev.

[B12] Zablotska IB, Gray RH, Serwadda D, Nalugoda F, Kigozi G, Sewankambo N, Lutalo T, Mangen FW, Wawer M (2006). Alcohol use before sex and HIV acquisition: a longitudinal study in Rakai, Uganda. AIDS.

[B13] Weiser SD, Leiter K, Heisler M, McFarland W, Percy-de Korte F, DeMonner SM, Tlou S, Phaladze N, Iacopino V, Bangsberg DR (2006). A population-based study on alcohol and high-risk sexual behaviors in Botswana. PLoS Med.

[B14] Fritz K, Woelk G, Bassett M, McFarland W, Routh J, Tobaiwa O, Stall R (2002). The association between alcohol use, sexual behavior and HIV infection among men attending beer halls in Harare, Zimbabwe. AIDS and Behavavior.

[B15] Kaljee LM, Genberg BL, Minh TT, Tho LH, Thoa LTK, Stanton B (2005). Alcohol use and HIV risk behaviors among rural adolescents in Khan Hoa Province, Viet Nam. Health Educ Res.

[B16] Stueve A, O'Donnell LN (2005). Early alcohol initiation and subsequent sexual and alcohol risk behaviors among urban youths. Am J Public Health.

[B17] Mataure P, McFarland W, Fritz K, Kim A, Woelk G, Ray S, Rutherford G (2002). Alcohol use and high risk sexual behavior among adolescents and among young adults in Harare, Zimbabwe. AIDS and Behavior.

[B18] Pillon SC, O'Brien B, Piedra Chavez KA (2005). The relationship between drugs use and risk behaviors in Brazilian university students. Rev Lat Am Enfermagem.

[B19] Deardorff J, Gonzales NA, Christopher FS, Roosa MW, Millsap RE (2005). Early puberty and adolescent pregnancy: the influence of alcohol use. Pediatrics.

[B20] Kebede D, Alem A, Mitike G, Enquselassie F, Berhane F, Abebe Y (2005). Khat and alcohol use and risky sex behaviour among in-school and out-of-school youth in Ethiopia. BMC Public Health.

[B21] Hughes TL (2005). Alcohol use and alcohol-related problems among lesbians and gay men. Annu Rev Nurs Res.

[B22] Gibney L, Saquib N, Metzger J (2003). Behavioral risk factors for STD/HIV transmission in Bangladesh's trucking industry. Soc Sci Med.

[B23] Ostermann J, Sloan FA, Taylor DH (2005). Heavy alcohol use and marital dissolution in the USA. Soc Sci Med.

[B24] Karlamangla A, Zhou K, Reuben D, Greendale G (2006). Longitudinal trajectories of heavy drinking in adults in the United States of America. Addiction.

[B25] National Institute of Statistics (NIS) and ORC Macro (2004). *Cameroon Demographic and Health Survey 2004*.

[B26] World Health Organization Global Status Report on Alcohol 2004. Geneva Switzerland.

[B27] Mennen LI, Mbanya JC, Cade J, Balkau B, Sharma S, Chungong S, Cruickshank JK (2000). The habitual diet in rural and urban Cameroon. Eur J Clin Nutr.

[B28] Eide AH, Acuda SW (1996). Cultural orientation and adolescents' alcohol use in Zimbabwe. Addiction.

[B29] Kongnyuy EJ, Wiysonge CS, Mbu RE, Nana P, Kouam L (2006). Wealth and sexual behaviour among men in Cameroon. BMC Int Health Hum Rights.

[B30] Gwatkin DR, Rustein S, Johnson K, Pande R, Wagstaff A, For the HNP/Poverty Thematic Group of the World Bank (2000). Socio-economic differences in health, nutrition and population in Cameroon.

[B31] Mitsunaga TM, Powell AM, Heard NJ, Larsen UM (2005). Extramarital sex among Nigerian men: polygyny and other risk factors. J Acquir Immune Defic Syndr.

[B32] Bishai D, Patil P, Pariyo G, Hill K The Babel effect: community linguistic diversity and extramarital sex in Uganda. AIDS Behav.

[B33] Buunk B (1980). Extramarital sex in the Netherlands: motivations in social and marital context. Journal of Family and Economic Issues.

[B34] Rissel CE, Richters J, Grulich AE, de Visser RO, Smith AM (2003). Sex in Australia: attitudes towards sex in a representative sample of adults. Aust N Z J Public Health.

[B35] Tveit KS, Nyfors A, Nilsen A (1996). Casual sex, extramarital sex, condom use and alcohol intake among heterosexual patients attending an STD clinic in Norway. Acta Derm Venereol.

[B36] Kimuna S, Djamba Y (2005). Wealth and extramarital sex among men in Zambia. Int Fam Plan Perspect.

[B37] Wiederman M (1997). Extramarital sex: prevalence and correlates in a national survey. Journal of Sex Research.

[B38] Ndubani P, Hojer B (2001). Sexual behavior and sexually transmitted diseases among young men in Zambia. Health Policy and Planning.

[B39] Ali MM, Cleland JG (2001). The link between postnatal abstinence and extramarital sex in Cote d'Ivoire. Studies in Family Planning.

[B40] Sudaryo MD, Detels R (1996). High risk behavior related to HIV transmission among recently diagnosed TB patients in Jakarta. Southeast Asian J Trop Med Public Health.

[B41] Atkins DC, Baucom DH, Jacobson NS (2001). Understanding infidelity: correlates in a national random sample. J Fam Psychol.

[B42] Cashdan E (1996). Women's mating strategies. Evolutionary Anthropology.

[B43] Bradford Hill A (1965). The environment and disease: association or causation. Proc R Soc Med.

[B44] Rashad I, Kaestner R (2004). Teenage sex, drugs and alcohol use: problems identifying the cause of risky behaviors. Journal of Health Economics.

[B45] Kapiga SH, Lyamuya EF, Lwihula GK, Hunter DJ (1998). The incidence of HIV infection among women using family planning methods in Dar es Salaam, Tanzania. AIDS.

